# Relations between Structure and Zn(II) Binding Affinity Shed Light on the Mechanisms of Rad50 Hook Domain Functioning and Its Phosphorylation

**DOI:** 10.3390/ijms231911140

**Published:** 2022-09-22

**Authors:** Józef Ba Tran, Michał Padjasek, Artur Krężel

**Affiliations:** Department of Chemical Biology, Faculty of Biotechnology, University of Wrocław, Joliot-Curie 14a, 50-383 Wrocław, Poland

**Keywords:** DNA damage, Rad50, zinc, zinc hook, Mre11-Rad50-Nbs1 (MRN)

## Abstract

The metal binding at protein–protein interfaces is still uncharted territory in intermolecular interactions. To date, only a few protein complexes binding Zn(II) in an intermolecular manner have been deeply investigated. The most notable example of such interfaces is located in the highly conserved Rad50 protein, part of the Mre11-Rad50-Nbs1 (MRN) complex, where Zn(II) is required for homodimerization (Zn(Rad50)_2_). The high stability of Zn(Rad50)_2_ is conserved not only for the protein derived from the thermophilic archaeon *Pyrococcus furiosus* (log*K*_12_ = 20.95 for 130-amino-acid-long fragment), which was the first one studied, but also for the human paralog studied here (log*K*_12_ = 19.52 for a 183-amino-acid-long fragment). As we reported previously, the extremely high stability results from the metal-coupled folding process where particular Rad50 protein fragments play a critical role. The sequence–structure–stability analysis based on human Rad50 presented here separates the individual structural components that increase the stability of the complex, pointing to amino acid residues far away from the Zn(II) binding site as being largely responsible for the complex stabilization. The influence of the individual components is very well reflected by the previously published crystal structure of the human Rad50 zinc hook (PDB: 5GOX). In addition, we hereby report the effect of phosphorylation of the zinc hook domain, which exerts a destabilizing effect on the domain. This study identifies factors governing the stability of metal-mediated protein–protein interactions and illuminates their molecular basis.

## 1. Introduction

**Figure 1:**
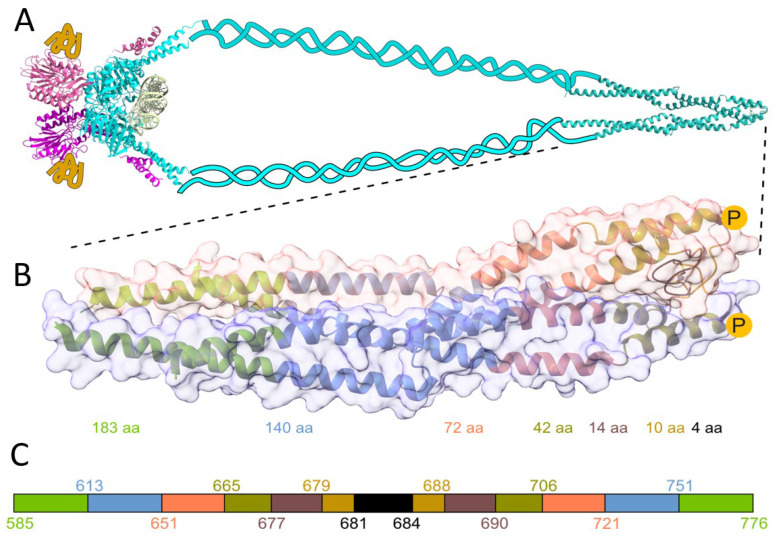
(**A**) Schematic architecture of the MRN complex. Light blue represents Rad50 dimer, pink and yellow structures, and ribbons represent two Mre11 and Nbs1 proteins, respectively. The structural representation of the globular domain of the MRN complex is based on the *Methanocaldococcus jannaschii* MR core complex bound to DNA (colored light green), (PDB: 5DNY) [20]. Rad50’s zinc hook from *H. sapiens* is shown magnified below. (**B**) Crystal structure of the investigated Rad50 protein fragment from *H. sapiens* (PDB: 5GOX) [19]. Zinc hook peptides and proteins (*Hs*Hk4-*Hs*Hk183) are used in the study to probe the effect of the interface on the Zn(*Hs*Hk)_2_-complex formation. The yellow circles with P in the middle show the site of phosphorylation of the Thr690 residue. (**C**) Schematic representation of peptides investigated here. The color-coded stretches and numbers represent the start and end of the fragments.

Metal ions are necessary for biological systems, fulfilling an enormous number of vital functions. To name only a few, metal ions provide ionic strength and are involved in basic metabolism, signaling, DNA synthesis, and DNA repair [1,2]. Given the omnipresence of metal ions in biological systems, it is no surprise that around 30–40% (estimated by bioinformatic means) of proteins in proteomes require one or more metal ions to accomplish their functions [3,4]. Despite various experimental methods that allow high-throughput identification of metalloproteins, the methods are still labor-intensive, making the description of the metalloproteome difficult even in the case of microorganisms, not to mention complex organisms like humans [5]. Among all d-block elements, zinc (formally Zn(II)) is the most common cofactor in proteins, occurring in up to 10% of proteins [6,7,8], thus making Zn(II)-binding proteins the most diverse and widespread group of metalloproteins [9]. Zn(II)-binding sites can be classified according to their function: catalytic, structural, regulatory, or transporting [10]. Another way of classifying Zn(II)-binding sites is based on the architecture of the site: the number and the type of coordinating residuals and number of coordinating polypeptide chains [11,12]. The vast majority of Zn(II)-binding metalloproteins bind their metal intramolecularly, i.e., a single polypeptide chain coordinating one or two Zn(II) ions, as is the case with zinc fingers. Less commonly but equally importantly, others bind Zn(II) ion(s) in an intermolecular way [13,14]. Intermolecular Zn(II) binding occurs when Zn(II) is bound to two or more polypeptide chains in a homo- or heteromolecular manner—the same or different protein components. Intermolecular Zn(II) binding sites have so far been described in terms of structure and function but rarely in terms of thermodynamic stability. One of the first stability-based descriptions of intermolecular Zn(II) binding sites is the analysis of the Rad50 Zn(II)-mediated protein dimer from *Pyrococcus* *furiosus* [1,15]. The dimerization is accomplished via the zinc hook domain formed by two antiparallel α-helices folded in half by a short β-loop hairpin with a highly conserved CXXC motif [1]. Here, the homodimeric Zn(II) complex formed by the Rad50s zinc hook domain from *Homo sapiens* is denoted as Zn(*Hs*Hk)_2_. The Rad50 protein is a component of the evolutionarily conserved MRN protein complex ((Mre11)_2_(Rad50)_2_(Nbs1)_2_/(Xrs2)_2_), playing a key role in the repair of DNA (Figure 1A). The complex is responsible for DNA double-strand breaks’ recognition, repair, and downstream signaling, and maintaining the correct telomere length [16,17,18]. Both ends of Rad50 N- and C-terminal domains with Walker A and B motifs form together the “head” region, which interacts with Mre11. Together with Mre11, Rad50 forms the heterotetrametric core complex Mre11_2_Rad50_2_, which in Eukaryotes is additionally enriched by interaction with nibrin (Nbs1) and Xrs2 in *Saccharomyces cerevisiae*), forming the Mre11_2_Rad50_2_Nbs1_2_(Xrs2_2_) complex [17]. The apex of the human monomer Rad50 protein takes the shape of a rod, with two alpha-helices forming a superhelical tertiary structure (Figure 1B) [12,19].

**Figure 2:**
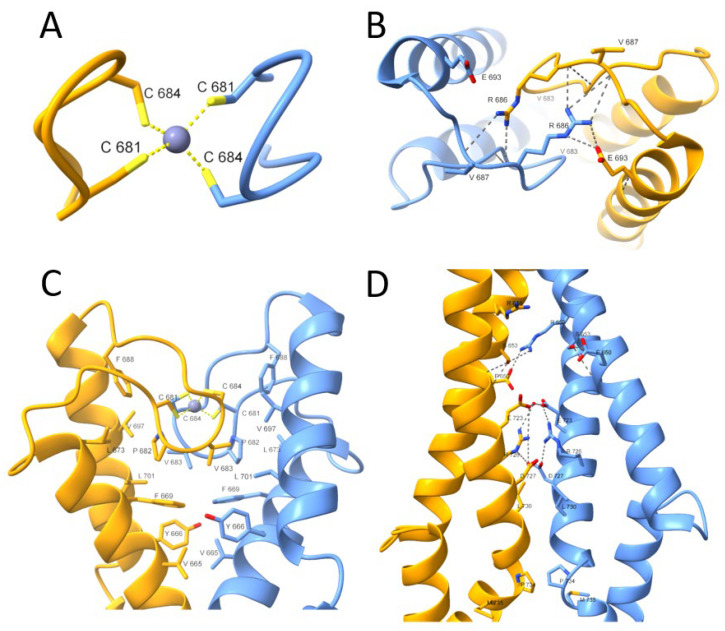
Close-up view of the *H. sapiens* Rad50 zinc hook domain (PDB: 5GOX) [19]. The presented interfaces stabilize the homodimeric structure of two Rad50 protomers. (**A**) The first interface is formed by Zn(II) coordination through the conserved CXXC motif. (**B**) The second interface stabilizes the Rad50 zinc hook domain and consists of ion pair interactions (Arg686-Glu693) and a hydrogen bond (Arg686-Val687). (**C**) The extensive network of hydrophobic interactions just below the second interface forms the third interface. (**D**) The last interface is formed at some distance from the rest, and its center is formed at the periphery by hydrophobic interactions and in the center by ionic pairs.

The Rad50 protein dimer is stabilized by several differentiated (varying in the type of the interaction) interfaces shown in Figure 2. The first interface is located in the very center of the superhelical structure, intersected by a β-hairpin containing the Zn(II)-binding motif (CXXC), called here a minimal zinc hook [1,19,21,22]. The Rad50 protein dimer is formed by binding the Zn(II) to two cysteinyl pairs from the CXXC motif, thus building an intermolecular Zn(II) complex called the zinc hook [15,19]. The complexation of Zn(II) by the CXXC motif forms the first interaction interface in Rad50 (Figure 2A). The importance of the CXXC motif is observable after cysteine to glycine residues’ mutation. Disruption of Zn(II)-binding to the zinc hook is lethal when exposed to ionizing radiation [1], which only underscores the fact that, similarly to Rad50, the zinc hook motif itself is conserved in all domains of life and even in some viruses [12,23]. The second interface (Figure 2B) stabilizes the first interface by forming an ion pair formed by Arg686 and Glu693 and a hydrogen bond with Arg686 and Val687. Further stabilization is formed by the third interface (Figure 2C), consisting mainly of hydrophobic residues. The last known interface based on PDB: 5GOX is formed by the ion pairs in the middle and hydrophobic interactions at the edge of the interface (Figure 2D) [19].

Even minor destabilization of the zinc hook domain significantly affects the long-range allosteric effect on the distant globular domain of Rad50, affecting all functions and Rad50’s complex stability [15,24]. The zinc hook domain is not only responsible for the proper functioning of Rad50 protein, but also long superhelical fragments that spatially separate the zinc hook domain from the ATPase domain. In spite of a small structural insight into the behavior of superhelical Rad50 fragments, their role is still elusive. It is known that the shortening of superhelical fragments of Rad50 protein negatively affects the occurrence of non-homologous end joining (NHEJ), while the disruption of DNA repair through homologous recombination occurs only when superhelical fragments are significantly shortened [25,26]. On the other hand, the ATP binding is disturbed even when a small superhelical fragment is removed [19]. The effect of either zinc hook or superhelical fragment damage on the functioning of the entire MRN complex is quite well understood [26], which indicates long-distance allostery between the zinc hook and globular ATP binding domains, giving superhelical fragments a prominent role in terms of signal transfer from one fragment of protein to another [16,26].

The architectural organization of Rad50 coiled coils has attracted much attention in recent years. Initially, the superhelical domains were thought to be just a flexible connector between the zinc hook and the globular ATPase domain. The emerging hypothesis is that Rad50’s coiled coils undergo structural transitions in the ATPase cycle and switch between an open, V-shaped-like conformation and a rod-shaped conformation. The existence of two differently orientated crystal structures, PDB: 1L8D [1] and PDB: 5GOX [19], seemed to confirm that assumption. However, the origin of the two structures is from two different species. Nevertheless, the most recent Rad50 structure from *P. furiosus* (PDB: 6ZFF) [21] confirms the existence of the rod-shaped zinc hook in a single organism. However, the structure published by Soh et al. does not explain the previously observed structure–complex stabilization behavior, where the stabilization increase occurs only in the ~45 amino acid long interacting fragment depicted in the V-shaped conformation [15,27]. Thus, to be able to say more about the influence of the rod-shaped conformation on Rad50, we decided to investigate the human Rad50 paralogue. Based on the data demonstrating ATP binding by Rad50 with truncated coiled coils, we hypothesize that shortening the Rad50 fragment will destabilize human Rad50’s Zn(II)-binding properties [19]. We were able to investigate the structural and energetic impact of the removed fragment on the complex formation by symmetrical shortening of the Rad50 zinc hook fragment from both N- and C- termini, as has been done similarly in the case of Rad50 from thermophilic *P. furiosus* [15,27].

An additional factor investigated for influencing Zn(II) binding properties was zinc hook domain phosphorylation. As has been shown by high-throughput studies, the Rad50 is phosphorylated at two sites: Ser635 and Thr690 [28,29,30]. While phosphorylation at Ser635 has been identified as necessary for subsequent ATM-dependent (ataxia-telangiectasia mutated kinase) signaling, the function of phosphorylation at Thr690 is still enigmatic. Regarding location, Ser635 is sited in the superhelical fragment of Rad50, while Thr690 is located at the apex of the zinc hook, near the Zn(II) binding site. Phosphorylation of the protein introduces a charged hydrophilic group in the polypeptide chain, which by modifying interactions with adjacent amino acid residues may change the protein conformation, protein complex stability, and formation, especially if the phosphorylation occurs near or at the binding interface. We have hypothesized that phosphorylation at Thr690 would change the complex stability of the Zn(Rad50)_2_ complex. Phosphorylation plays a key role in fine-tuning many cellular processes such as the cell cycle, apoptosis, growth, and signal transduction pathways, to name only a few [31]. Here, we examined biophysical properties of Rad50 such as Zn(II)-to-protein affinity (called here stability), structural flexibility, and conformational changes occurring upon Zn(II) binding using different biophysical methods. Based on the known human Rad50 zinc hook structure, we investigated the properties of small peptides, from four-amino-acid-long, through 42-amino-acid-long phosphorylated and phosphomimetic mutant peptides (as a model for phosphorylated counterpart) and longer fragments, up to 183-amino-acid protein fragments (Figure 1C, Table S1).

## 2. Results

Previous studies on the zinc hook domain from *P. furiosus* showed that Zn(II) is necessary to form a highly stable Rad50 homodimer [15,19]. Close sequence–structure analysis showed that such high stability is governed by the unique domain folding occurring upon Zn(II) binding and the subsequent formation of numerous electrostatic and hydrophobic interactions in the domain that significantly elevate the thermodynamic stability of the Zn(Cys)_4_ core that exists between two Rad50 protomers [15,22]. Detailed analysis of structural elements that participate in overall stability was facilitated by studying the hook domain using *Pf*Hk peptide models of Rad50 fragments from its apex from 4 to 130 amino acid residues of various lengths [15,27]. To investigate relations between structural, thermodynamic, and stability properties of human Rad50, we applied the same strategy and used peptides ranging in length from 4 to 72 amino acid residues (designated as *Hs*Hk4-*Hs*Hk72) and two protein constructs containing 140 and 183 amino acid residues (*Hs*Hk140 and *Hs*Hk183, respectively) (Figure 1C, Table S1). Peptides ranging in length from 4 to 42 were designed analogously to model *Pf*Hk peptides to contain two N- and C- termini of similar length [15]. The need to investigate longer fragments is derived from the fact that the *Hs*Hk42 model peptide from *H. sapiens*, contrary to the model peptide from *P. furiosus* of similar length (*Pf*Hk45), does not contain all intermolecular contacts in the complex, thus requiring us to prepare longer fragments. To probe the impact of Thr690 phosphorylation on zinc hook stability, we synthesized phosphorylated *Hs*Hk42 (*Hs*Hk42 pT690) and phosphomimetic *Hs*Hk42 (T690E) peptides and prepared a phosphomimetic variant of *Hs*Hk183 (*Hs*Hk183 T690E) protein (Table S1).

### 2.1. Folding of the Hook Domain Induced by Zn(II)

**Figure 3:**
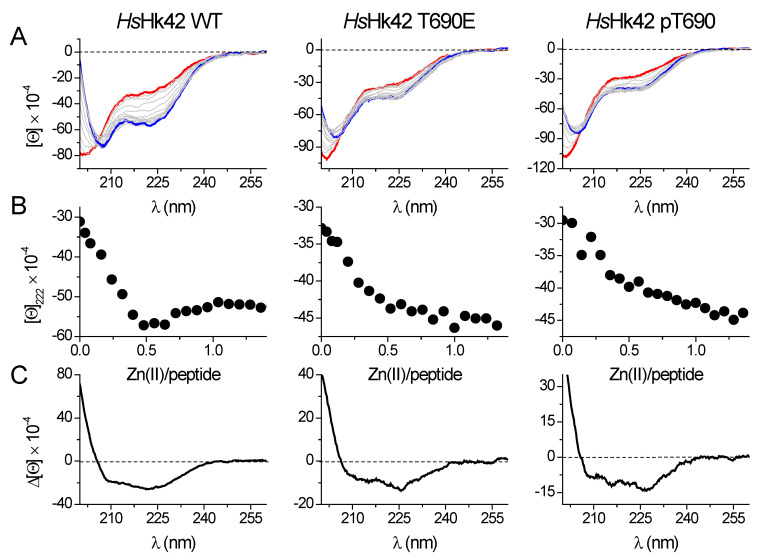
Zn(II) binding to 42-amino-acid long *Hs*Hk peptides registered by spectropolarimetry in 20 mM Tris-HCl buffer, pH 7.4, 100 mM NaF. (**A**) CD spectra of the apo-form of the peptides are shown as a red line, and the complex at a 0.5 molar ratio is shown as a blue line. (**B**) Molar ellipticity changes measured at 222 nm in the function of Zn(II)/peptide molar ratio. (**C**) Differential CD spectra for *Hs*Hk42 peptides were obtained by subtracting the apo-form spectra from the spectra of complexes at a 0.5 molar ratio. [Θ] corresponds to molar ellipticity in deg × cm^2^ × dmol^−1^.

The binding of Zn(II) to model peptides was monitored using circular dichroism spectroscopy (CD) by the titration of ZnSO_4_ to apo-peptides at pH 7.4 in the presence of the low affinity metal binding reducing agent TCEP [32]. A sharp inflection characterizes almost all titration curves at a molar ratio of 1:2 Zn(II)-peptide, as all investigated peptides form Zn(II)-mediated homodimers, ZnL_2_ (Figure S1). Only *Hs*Hk10 and *Hs*Hk14 do not present sharp inflection at this Zn(II)-peptide ratio, suggesting that besides ZnL_2_ species, the ZnL complex (1:1 Zn(II)-peptide ratio) is also formed during titration. The shape of the CD spectra is determined by the secondary structure adopted by the peptide or protein and varies depending on the studied *Hs*Hk peptide/protein. Structural changes provoked by Zn(II) binding and thus the formation of the Zn(*Hs*Hk)_2_ complex can be compared qualitatively by differential spectra, obtained by subtracting the apo-form (metal-free) spectrum from the holo-form (Zn(II)-saturated) spectrum. The length of the peptides determines the spectra features, i.e., shorter peptides (*Hs*Hk4-*Hs*Hk14) represent spectra more similar to those for peptides with β-structures (Figure S1). The longer peptides share spectrum characteristics for α-helix-containing peptides with two minima at 208 and 222 nm. Moreover, this characteristic increases with peptide length from *Hs*Hk42 to *Hs*Hk183, indicating an increase in the amount of α-helix in formed Zn(II) complexes. The 222 and 208 nm ratio confirms the increased coiled-coil structure fraction [33]. Zn(II) induced folding of investigated Rad50 fragments may suggest that the structurization effect propagates over the whole coiled-coil segment up to globular domains, which possibly could explain why disruption of the Zn(II) binding motif results in loss of some of the MRN functions [25,33] and why point mutations may impair Rad50 functions [19]. Posttranslational modifications are in some respects similar to point mutations; they are local, yet they may influence the global behavior of the single protein or protein complex. These modifications, e.g., phosphorylation, may fine-tune or modify protein stability and protein function. We hypothesized that the phosphorylation at the apex of human Rad50’s zinc hook at Thr690 may somehow exert a similar effect. To test this hypothesis, we titrated phosphorylated *Hs*Hk42 (*Hs*Hk42 pT690) and its phosphomimetic counterpart, *Hs*Hk T690E, with Zn(II). The use of a phosphomimetic was motivated by use of a more extended protein model (*Hs*Hk183) in a further study, in which introducing the phosphorylated Thr690 could be cumbersome. As it turned out, both *Hs*Hk42 pT690 and *Hs*Hk42 T690E present similar, different from *Hs*Hk42, secondary structure conformational changes induced by Zn(II) binding, as indicated by CD titration (Figure 3). The structurization of *Hs*Hk42 pT690 and *Hs*Hk42 T690E peptides compared to *Hs*Hk42 is much smaller; in addition, the changes observed at 222 nm are also less visible, with the inflection at the 1:2 Zn(II)-peptide ratio not so well outlined as in the case of the *Hs*Hk42 peptide.

**Figure 4:**
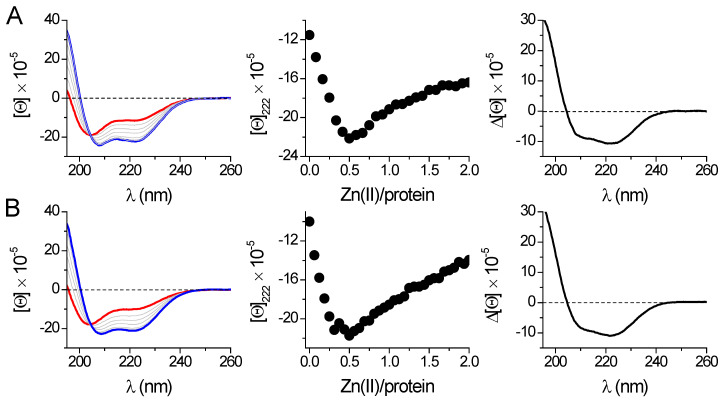
Zn(II) binding to 183-amino-acid-long proteins *Hs*Hk183 (**A**) and *Hs*Hk183 T690E (**B**) was monitored spectropolarimetrically in 20 mM Tris-HCl buffer, pH 7.4, 100 mM NaF. The left panel shows spectra of the apo-form (red line) titrated with Zn(II); the complex at a 0.5 molar ratio is shown as a blue line. The middle panel shows ellipticity changes at 222 nm in the Zn(II)/protein molar ratio function. The right panel presents differential CD spectra for *Hs*Hk183 protein fragments obtained by subtraction of the apo-form spectra from the spectra of complexes at a 0.5 molar ratio. [Θ] corresponds to molar ellipticity in deg × cm^2^ × dmol^−1^.

Contrary to 42-amino-acid-long peptide models, direct CD titrations of both metal-free *Hs*Hk183 and phosphomimetic *Hs*Hk183 T690E with Zn(II) show extensive conformational changes (Figure 4). Furthermore, the Zn(II) binding to protein fragments causes considerable changes in CD spectra, indicating metal-induced folding and coiled-coil stabilization. The measured spectra for *Hs*Hk42 peptides and *Hs*Hk183 proteins show that with the length of the structure, the effect of phosphorylation or phosphomimetic mutation on the secondary structure disappears. Nevertheless, the stability of the complex cannot be inferred from CD spectra alone. Therefore, we applied classical potentiometry and competitive titrations with metal chelators to assess the impact of phosphorylation on the stability of Zn(*Hs*Hk)_2_ complexes.

### 2.2. Stability of the Zinc Hook Domain

We used potentiometric (*Hs*Hk6 and *Hs*Hk10) and competitive CD titrations with zinc chelators, buffering Zn(II) in a broad free Zn(II) concentration range (CDTA, EDTA, HEDTA, EDDS, EGTA, NTA) for human Rad50 fragments (*Hs*Hk10–*Hs*Hk183) (Figure S2) to measure the stability of the Zn(II)-*Hs*Hk complexes [15].

The potentiometry was performed to determine the number of Zn(II)-*Hs*Hk species and their stability constants. Figure S3 presents the species distribution for both peptides under the conditions used in potentiometry. To compare the stability of the zinc hook domain, independently from Rad50 fragment length and method of the determination, we used the cumulative constant (*K*_12_) that describes the formation of the dimeric zinc hook complex (Zn(*Hs*Hk)_2_) given by Equation (1):

(1) $$\begin{array}{c} \mathrm{Zn}\left(\mathrm{II}\right)+2Hs\mathrm{Hk}\;⇋\;\mathrm{Zn}{\left(Hs\mathrm{Hk}\right)}_{2}\;\left(K_{12}=K_{1}\times K_{2}\right)\; \end{array}$$

(2) $$\begin{array}{c} \mathrm{Zn}\left(\mathrm{II}\right)+Hs\mathrm{Hk}\;⇋\;\mathrm{Zn}Hs\mathrm{Hk}\;\;\left(K_{1}\right)\;\;\;\;\; \end{array}$$

(3) $$\begin{array}{c} \mathrm{Zn}Hs\mathrm{Hk}+Hs\mathrm{Hk}\;⇋\;\mathrm{Zn}{\left(Hs\mathrm{Hk}\right)}_{2}\;\;\left(K_{2}\right)\;\;\;\; \end{array}$$

where Equations (2) and (3) represent the formation of Zn*Hs*Hk (ZnL) and Zn(*Hs*Hk)_2_ (ZnL_2_) complexes in a stepwise manner. Constant *K*_1_ is not always possible to determine, especially when the ZnL complex stability is low, and the 1:2 biscomplex is characterized by high stability (*K*_2_ ≪ *K*_1_). In such cases, the only observable form is ZnL_2_ species, allowing only for the determination of cumulative constant *K*_12_. The method of *K*_12_ value determination is described in Materials and Methods and previous reports [15,19,22,27]. However, it must be mentioned that *K*_12_ constants determined in competitive experiments were calculated from half saturation points (−log[Zn(II)]_free_) obtained by equilibration of *Hs*Hk peptides and proteins in metal buffers (partially saturated metal chelators with various Zn(II) affinities), as presented in Figure S2. The *K*_12_ values from cumulative protonation and stability constants (*β*_ijk_) from potentiometric titrations (Table S2) were calculated based on species distribution presented in Figure S3. All determined and calculated log*K*_12_ values are presented in Table 1.

**Figure 5:**
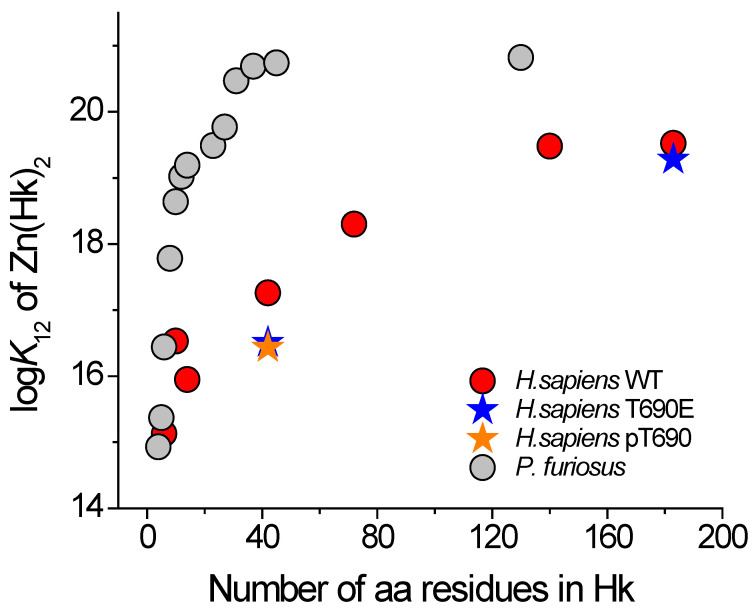
Dependence of conditional formation constants (log*K*_12_) of Zn(*Pf*Hk)_2_ (gray circles) and Zn(*Hs*Hk)_2_ (red circles) complexes at pH 7.4 on the length of zinc hook peptides [15,27]. Orange and blue stars correspond to T690E and pT690, respectively.

Data presented in Table 1 show that log*K*_12_ values for *Hs*Hk4–*Hs*Hk14 are relatively similar to each other, regardless of the method of determination. However, the values increase from 14.93 for *Hs*Hk4 [15] to 16.15 or 16.5 for *Hs*Hk10, indicating a length-dependent trend. However, the log*K*_12_ for *Hs*Hk14 is lower and reaches the value of 15.8. The longer human Rad50 fragments showed a substantial increase in complex stability, which is similar to the Rad50 paralog from *P. furiosus* [15]. This elevated complex stability is associated with a more pronounced formation of the helical and coiled-coil structures in investigated protein fragments. In the case of *Hs*Hk42, peptide phosphorylation (*Hs*Hk42 pT690) and phosphomimetic mutation (*Hs*Hk42 T690E) decrease complex stability by 0.8 and 0.7 orders of magnitude of log*K*_12_, respectively. This effect is transmitted to longer protein fragments, where phosphomimetic mutation decreases Zn(*Hs*Hk183)_2_ complex stability by 0.2 orders of magnitude, showing that elongation of model peptides increases the participation of the coiled-coil segment in complex stabilization (Table 1). This effect, despite length-dependent similarity, is quite different compared to previously published data for the *P. furiosus* zinc hook domain (Figure 5). The human Rad50 complex is weaker overall in terms of stability and complex stabilization, but above all, the stabilization occurs over longer fragments. The stabilization pattern of Zn(*Hs*Hk)_2_ complexes assumes a more linear form, whereas the stabilization of the zinc hook homodimer from *P. furiosus* (Zn(*Pf*Hk)_2_) adopts logarithmic growth. In the case of the zinc hook domain from *P. furiosus*, the stabilization occurs in two major regions. The first major region is in the minimal zinc hook (14-amino-acid-long β-hairpin fragment containing CXXC binding motif). Another stabilization event occurs by increasing the length of distal to minimal zinc hook polypeptide chains, up to around 45 amino acids long in total [15]. The further elongation of the polypeptide chain, up to 130 amino acids long, does not increase the Zn(*Pf*Hk)_2_ complex stability [27]. The stabilization pattern of the zinc hook domain from *H. sapiens* also shows stabilization in two regions, but in contrast to the zinc hook domain from *P. furiosus*, the two stabilization regions are intersected by a short destabilizing region; i.e., *Hs*Hk10 has a higher affinity towards Zn(II) than the longer *Hs*Hk14, by up to 0.7 orders of magnitude of log*K*_12_, as determined by competition studies. This short destabilization may be an effect of the introduction of a peptide fragment that is too short to be able to form stabilizing bonds by itself. *Hs*Hk10 compared to *Hs*Hk6 introduces the possibility of forming hydrogen bonds between Arg686 and Val687, but *Hs*Hk14 compared to *Hs*Hk10 does not introduce residues that could stabilize the interface. The stabilization that occurs by elongation beyond the length of 14 amino acids occurs consistently with the distribution of interfaces along the rod-shaped structure (Figure 2).

### 2.3. Small Angle X-ray Scattering (SAXS) Confirms Rod-Shaped Phosphomimetic Zinc Hook

**Figure 6:**
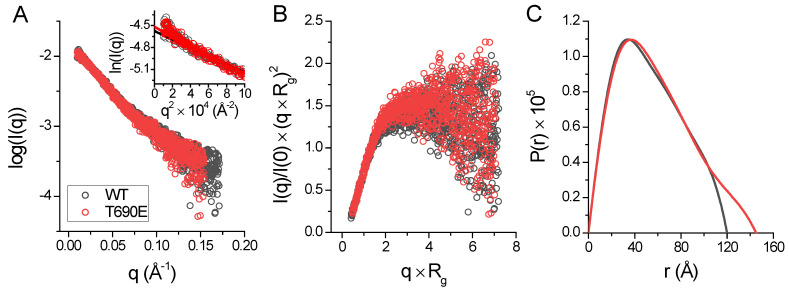
(**A**) X-ray scattering profile of *Hs*Hk183 (black colored) and *Hs*Hk183 T690E (red colored) in solution. The Guinier plot is shown in the insert. (**B**) Normalized Kratky plot for subjected samples is characteristic for partially unfolded or rod-shaped proteins. (**C**) Pair distance distribution P(r) function of *Hs*Hk183 (black colored) and *Hs*Hk183 T690E (red colored) is characteristic for rod-shaped particles [34].

To answer the question of whether phosphomimetic mutation induces conformational changes of *Hs*Rad50, we performed a size-exclusion chromatography (SEC)-SAXS experiment. Figure 6A shows the SAXS scattering profile for the proteins. For both proteins, the Kratky plot of the SAXS data shows scattering profiles similar to profiles of partially folded or rod-shaped proteins (Figure 6B) [33]. The *Hs*Hk183 and *Hs*Hk183 T690E have a similar radius of gyration, 44.584 Å and 45.956 Å, respectively, as well as the maximum dimension of the particle (D_max_) 118.9 Å and 132.1 Å.

**Figure 7:**
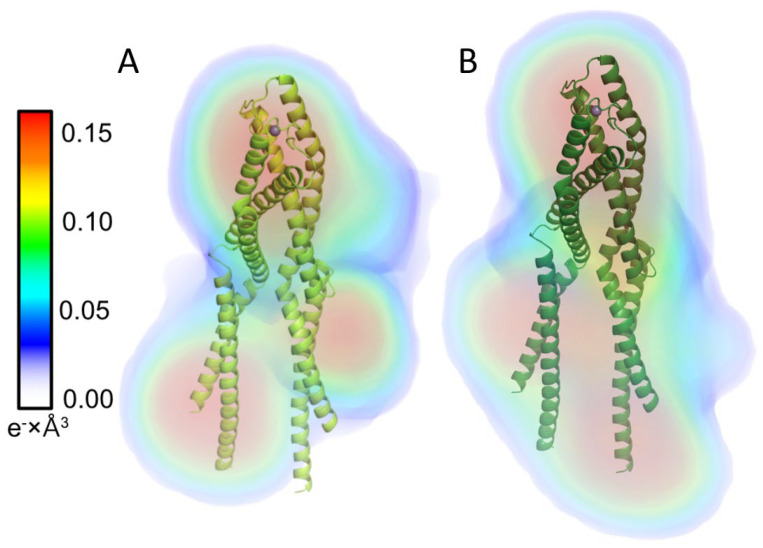
Electron density reconstruction from experimental solution scattering data for (**A**) *Hs*Hk183 and (**B**) *Hs*Hk183 T690E. Electron densities are shown as colored volumes. The color bar on the left indicates the electron density value in e^−^ × Å^3^. X-ray crystal structure of Rad50 protein fragment is presented in cartoon format (PDB ID 5GOX). *Hs*Hk183 and phosphomimetic mutant form rod-shaped particles.

For both proteins, the distance distribution function P(r) shows (Figure 6C) a single peak with an elongated tail, which is distinctive for an elongated structure [34]. Ab initio models generated with DENSS [35] for *Hs*Hk183 and *Hs*Hk183 T690E show a rod-shaped particle which is similar to the Rad50 crystal structure (Figure 7). These results, taken together, indicate that the conformations adopted by both *Hs*Hk183 and *Hs*Hk183 T690E in solution are similar to conformations adopted in the crystal (PDB 5GOX [19]), and the phosphomimetic mutation impact on the overall tertiary and quaternary structure is negligible, or not observable by low-resolution methods like SAXS.

### 2.4. X-ray Absorption Spectrum in the Near-Edge Structure and Extended X-ray Absorption Fine Structure Show Tetrathiolate Zn(II) in HsHk183

**Figure 8:**
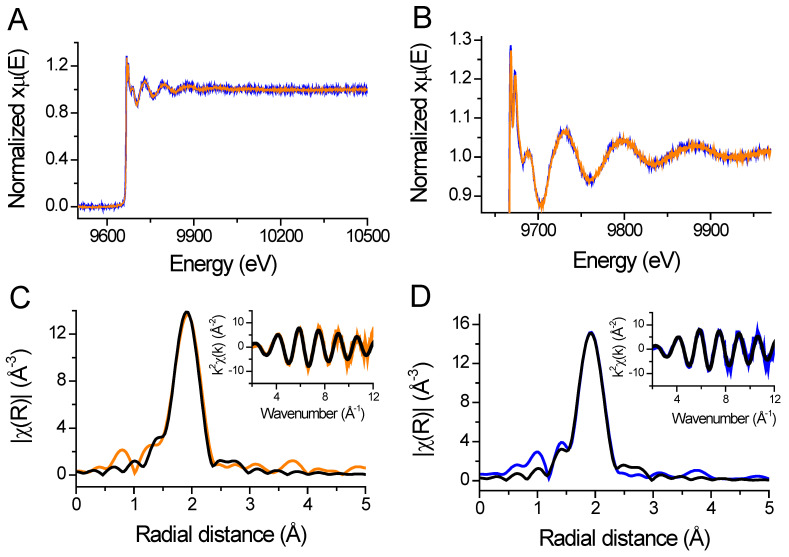
(**A**) Full X-ray absorption spectra of *Hs*Hk183 (orange line) and *Hs*Hk183 T690E (blue line) represented as normalized absorption coefficient plotted versus energy. (**B**) Zoomed in near edge region and extended absorption spectra. (**C**,**D**) Fourier-transformed and *k*^2^-weighted (insert) data for Zn(*Hs*Hk183)_2_ and Zn(*Hs*Hk183 T690E)_2_, respectively. The black line represents the fit to the data.

XANES is a structural technique that probes the local environment of a metal ion. The X-ray absorption spectrum in the near-edge structure (XANES) is responsive to changes in the coordination mode of the metal ion. Data collected at the K edge of the metal are sensitive to the effective charge and the coordination geometry, while the extended X-ray absorption fine structure (EXAFS) region of the spectrum can inform metal–ligand distances and the number of ligands [36]. We applied both XANES and EXAFS to test to determine whether phosphomimetic mutation at the apex of the zinc hook (*Hs*Hk183 T690E) induces local changes in the coordination environment of Zn(II) using *Hs*Hk183 as a control. The EXAFS spectra and their corresponding Fourier transforms of *Hs*Hk183 and *Hs*Hk183 T690E are shown in Figure 8. *Hs*Hk183 and *Hs*Hk183 T690E X-ray absorption spectra are almost identical (Figure 8A,B), with small differences due to noise. The data for proteins were analyzed in the range of k = 3–12 Å^−1^, which is typical for peptides or proteins [37]. The Fourier transforms for both samples show a first shell peak at a radial distance of 1.92 Å and smaller outer-shell peaks at a radial distance of 2.3–4.0 Å (Figure 8C,D). Fit to experimental data based on the crystal structure of human Rad50 (PDB 5GOX) [19] explains that the first-shell peak represents the scattering of 4 sulfur, placed ~2.3 Å from Zn(II); additionally, smaller shell features (observed at distances of R = ~3–5 Å) may arise from the single and multiple scattering of atoms such as carbon, nitrogen, and oxygen [36,38] or may be a Fourier transform truncation effect [37]. The results of fitting the EXAFS curve to experimental data are summarized in Table S3. For both proteins (*Hs*Hk183 and *Hs*Hk183 T690E), the quality of the fits is good (χ^2^*_Hs_*_Hk183_ = 45.65 and χ^2^ *_Hs_*_Hk183 T690E_ = 52.85 with low R-factor values of 7.84 × 10^−3^ and 8.25 × 10^−3^, respectively, for *Hs*Hk183 and *Hs*Hk183 T690E) and explains the observed spectra. Both XANES and EXAFS features confirm that the Zn(II) coordination in protein is tetrathiolate, and phosphomimetic mutation does not influence the local coordination geometry of the Zn(II) in the 183-amino-acid-long Rad50 zinc hook domain.

## 3. Discussion

Specific protein–protein interactions are usually stabilized by an extensive network of non-covalent intermolecular interactions—their joint energy effect must outweigh the adverse energy effect associated with entropy decrease and dehydration of protein surfaces [39,40]. An additional factor contributing to the formation of protein complexes is intermolecular metal binding [13,41,42]. In such a case, the affinity of the metal for the protein will affect the stability of the entire complex [13,43]. For example, among the classical factors that affect the affinity of the Zn(II) complex are acid–base properties of Zn(II) ligands in structural sites, first coordination sphere composition, stabilization of ligands coordinating Zn(II), and metal-coupled folding of proteins [12,44,45,46,47]. In this respect, the interaction present in the zinc hook domain of the human Rad50 protein is unique and so far not observed anywhere else in nature—the stability of the Zn(II)-Rad50 complex is also influenced by structuration that is distant from the coordination site. Similar studies regarding the stability of complexes with structural zinc binding sites (e.g., zinc fingers) depending on investigated length show similar behavior of elevated stability; nevertheless, none of those reports show stabilization over such an extent, thus making the human Rad50 zinc hook domain unique and the first known complex to behave in such a manner [48,49,50].

The current study provides a detailed analysis of the stabilization of the human Rad50 hook domain. We anticipated that elongation of the investigated peptides would increase the stability of the complex by increasing the number of inter- and intra- interactions. Stabilization of the zinc hook domain isolated from *P. furiosus* occurs primarily in a small 45-amino-acid-long fragment, where all present residues participate in the domain stabilization. Elongating the fragment above 45 amino acid residues does not impact the domain stability. In contrast, human Rad50 zinc hook domain stabilization occurs over a long peptide fragment of up to 140 amino acid residues. As the occurrence of intermolecular Zn(II) binding sites is rather rare (or has a low discovery rate), our knowledge of the stability of such sites is very limited. To date, only a few intermolecular binding sites have been examined for Zn(II) affinity. For example, intermolecular Zn(II) binding sites in CD4/CD8a co-receptors—Lck kinase complexes [3,51,52]—and a Rad50 homolog from *P. furiosus* have been investigated in terms of stability [15]. On the other hand, Zn(II) affinity comparison of a complex that contains intermolecular Zn(II) with complexes with intramolecular Zn(II) (e.g., zinc fingers) is definitely more difficult due to differences in stoichiometry and constants’ definition, but can be done by applying the competitivity index (CI) [9,53], as demonstrated in Table 2. This type of analysis shows that human Rad50 far outperforms Zn(II) ions in binding strength over the strongest zinc fingers. The effect of Zn(II) transfer from zinc fingers to the Rad50 protein would also be observable in the phosphorylated or phosphomimetic Rad50 protein, where there is a reduction in binding affinity by as much as one-fourth of an order of magnitude (in the case of *Hs*Hk183 and *Hs*Hk183 T690E). It seems that phosphorylation induces local changes just at the Zn(II) binding motif, which is observable during direct Zn(II) titration of *Hs*Hk42 peptides with spectropolarimetric detection; however, in the case of direct titration of *Hs*Hk183, these changes are masked by a large contribution of the signal coming from the superhelical fragment. Additionally, these changes are not large enough to be observed with low-resolution methods like SAXS. We hypothesized that the structural change induced by phosphorylation would be observable in the first coordination sphere of the Zn(II) bound by the CXXC motif. To investigate this, we applied X-ray absorption spectroscopy. Both XANES and EXAFS have been used previously to examine the structure of Zn(II) binding sites of various proteins, e.g., serum albumin [54], alkaline phosphatase [36], alcohol dehydrogenase [55], HIV-2 integrase [56], carboxypeptidase A [57], protein farnesyltransferase [58], DNA primase from bacteriophage T7 [59], and others. Additionally, phosphorylation also does not induce changes in the direct Zn(II) coordination sphere, as observed with EXAFS. The fact that we have not observed local (EXAFS) and global structural changes (SAXS) in *Hs*Hk183 constructs does not necessarily mean that the phosphorylation does not influence conformational changes of Rad50. Potentially, these changes could be observed by other methods, e.g., fluorescence resonance energy transfer or crystallization.

Lowering the affinity of Zn(II) for a protein even by one-fourth of an order of magnitude is a substantial effect. However, in the case of the Rad50 protein, the binding is so strong that in the cell the Rad50 protein is virtually constantly saturated with Zn(II), possibly excluding the phosphorylation-dependent regulation of the Zn(II) binding site. Rad50 phosphorylation at threonine 690 may be involved in as yet undiscovered signaling pathways, similar to how phosphorylation at serine 635 is engaged in ATM-dependent downstream signaling for DNA repair and cell cycle control [63].

The sequence alignment of the studied *H*. *sapiens* Rad50 zinc hook fragments with the corresponding fragments from *P. furiosus* show decreasing sequence similarity (Figures S4 and S5). Although the fragments differ in sequence, they are characterized by a superior complex stability. What accounts for such high affinity? In the case of the zinc hook complex from *P. furiosus*, the answer seems obvious—the amino acid moieties involved in forming the intermolecular interface are identified in the crystal structure of the complex (PDB: 1L8D) [1]. The crystal structure of 1L8D shows the zinc hook domain, in which two monomers dimerize at the interface through a Zn(II) binding motif, interacting over a length of about 45 amino acids (forming a second interface immediately after the Zn(II) binding motif itself), and then diverge without contacting lower down. This model is confirmed by examining the stability of a 130-amino-acid hook from *P. furiosus*—the stability of the hook does not increase with increasing length beyond 45 amino acid residues, which indicates that there is no major interface in the 130 amino acid fragment [15,27]. Chimeric Rad50 protein, with the zinc hook from *P. furiosus* and the superhelical fragment from *H. sapiens*, maintains an open conformation, with laterally spaced superhelical fragments, as verified by SAXS [19]. Although it seemed that the extensive superhelical interface in the Rad50 protein is restricted to eukaryotic homologs, this view has changed with recent discoveries. Recent work regarding the structure of the zinc hook domain of the Rad50 protein from *P. furiosus* (PDB: 6ZFF) [21] also shows a closed conformation with adjacent superhelical fragments, in which the intermolecular interface is formed by the contact of two amino-terminal α-helices, crossing at approximately 60°, in close proximity to the zinc-binding motif (20 Å). This interface extends along the amino-terminal α-helix, although closer to the end the interface is decidedly less outlined and presents greater distances between amino acid residues (Figure S6). The reduced contacts in this part of the protein may not necessarily be the actual behavior of the protein in solution but are present due to packing interactions in the crystal [13,21].

A comparison of the two crystal structures of the rod-shaped Rad50 protein shows that there are four main interfaces of interaction in the rod-type structure of the human Rad50 protein (PDB ID 5GOX): (1) the CXXC binding motif, (2) the vicinity of the CXXC motif, (3) about 20 Å away from the Zn(II), the α-terminal helices crossing at an angle of about 60°, (4) and an interface about 60 Å away from the Zn(II) formed by the carboxy-terminal α-helices (Figure 1, Figure S6). As described above, the poorly defined third interface in the case of the rod structure of the *P. furiosus* Rad50 homolog is probably due to the packing interactions occurring in the crystal. Within the third interface of *H. sapiens*, the amino-terminal helices form loops that are absent in the case of the *P. furiosus* paralog; nevertheless, both conformations present similar architecture of the complexes. The rod-shaped model of the human Rad50 protein fully explains the increase in stability of the Zn(*Hs*Hk)_2_ complex correlated with symmetric elongation of the domain. Elongation of the peptides to form the described interfaces further increases the stability of the zinc hook; however, after reaching a certain length, containing all three interfaces, further elongation (up to *Hs*Hk183) does not result in a stability gain. This indicates that the amino acid residues contained in *Hs*Hk140 are largely responsible for the affinity of Zn(II) to human Rad50 and shows that amino acid residues well away from the Zn(II) binding site can have a large effect on the affinity of the protein for the metal.

The recently described, rod-shaped structure of the Rad50 protein [21] from *P. furiosus* does not explain the observed relationship between stability and length of the previously investigated fragment. If the rod/closed structure were indeed the dominant form in the solution, the stability increase should be observed in the fragment responsible for forming the interface about 20 Å and 60 Å away from the zinc hook; however, this is not the case, as the *Pf*Hk45 and *Pf*Hk130 fragments tested have comparable stability [15,27]. The observable effect could be explained by the equilibrium—it is possible that the investigated zinc hook domain fragments from *P. furiosus* exist in equilibrium between the rod form and the open form, with the equilibrium shifted towards the open form. If this is the case, then the sum effect observed by competitive titration with detection by circular dichroism will depend primarily on the presence of the open form. The residual population of molecules that form the rod conformation could explain the crosslinking pattern with a short (8 Å) bis-maleimidoethane (BMOE) crosslinker, indicating that a rod-shaped form is present in the solution [21]. This type of crosslinking pattern would not be seen if only the open structure were present in the solution. It seems that Rad50 may adopt different conformations depending on its current state of DNA damage repair [64,65]. A recent study on the homolog of the MR complex from *E. coli*, the SbcCD complex, shows the complex in different conformations: cutting-state and resting-state [18]. The transition from the closed conformation to the open one is probably connected with a high energy cost resulting from the necessity to break numerous interactions at the interface.

This study presents a thorough analysis of the factors responsible for the stabilization of the human Rad50 protein dimer with a Zn(II) bound at the protein–protein interface using model peptides with gradually increasing lengths. Although the study mainly focused on the stability of human Rad50 protein, it provides insight into the mechanisms of stabilization in Rad50 from *P. furiosus*, suggesting that the major form in solution is the open form. Unlike the *P. furiosus* protein, the stabilization of the human Rad50 protein is fully explained by the rod-type structure. The stabilization pattern of the human Rad50 protein is quite different from that of the *P. furiosus* protein. In the case of the latter, the largest stabilization effect occurs at the small central β-splice fragment, whereas in the case of the human Rad50 protein, the stabilization effect is more distributed, proceeds in a more linear fashion, and strongly shows the large effect of coiled-coil fragments on stabilization. The destabilization of the zinc hook from human Rad50 induced by the phosphorylation of threonine 690 is significant, although with physiological availability of Zn(II) in the cell, it does not cause dissociation of the metal from the protein, suggesting that the phosphorylation does not regulate the MRN complex by orchestrating the Zn(II) saturation of Rad50, but rather by a still unknown pathway.

## 4. Materials and Methods

### 4.1. Materials

All Fmoc-protected amino acids for peptide synthesis (Fmoc-Ala-OH∙H_2_O, Fmoc-Arg(Pbf)-OH, Fmoc-Asn(Trt)-OH, Fmoc-Asp(OtBu)-OH, Fmoc-Cys(Trt)-OH, Fmoc-Gln(Trt)-OH, Fmoc-Ile-OH, Fmoc-Leu-OH, Fmoc-Lys(Boc)-OH, Fmoc-Met-OH, Fmoc-Phe-OH, Fmoc-Pro-OH, Fmoc-Ser(tBu)-OH, Fmoc-Thr(tBu)-OH, Fmoc-Tyr(tBu)-OH, Fmoc-Val-OH), piperidine, 3-[bis(dimethylamino)methyliumyl]-3*H*-enzotriazol-1-oxide hexafluorophosphate (HBTU), *N,N*-diisopropylethylamine (DIEA), trifluoroacetic acid, tris(2-carboxyethyl)phosphine hydrochloride (TCEP) were obtained from Iris Biotech GmbH (Marktredwitz, Germany). Acetic anhydride was obtained from Avantor Performance Materials Poland (Gliwice, Poland). TentaGel R RAM resin for peptide synthesis was obtained from Rapp Polymere GmbH (Tübingen, Germany). Phenol, thioanisole, anisole, triisopropylsilane (TIPS), guanidine hydrochloride (GdmHCl), trans-1,2-diaminocyclohexane-*N,N,N′,N′*-tetraacetic acid monohydrate (CDTA), ethylenediaminetetraacetic acid (EDTA), bis(βaminoethyl ether)-*N,N,N′,N′*-tetraacetic acid (EGTA), *N*-carboxymethyl-*N′*-(2-hydroxyethyl)-*N,N′*-ethylenediglycine (HEDTA), ethylenediamine-*N,N′*-disuccinic acid (EDDS), 2,2′,2″-nitrilotriacetic acid (NTA), HCl (trace metal grade), NaClO_4_·H_2_O, ZnSO_4_·7H_2_O and 3CdSO_4_·H_2_O were purchased from Merck KGaA (Darmstadt, Germany). DL-dithiothreitol (DTT), 4-(2-hydroxyethyl)piperazine-1-ethanesulfonic acid sodium salt (HEPES), tryptone, yeast extract, and LB medium were bought from Bioshop (Burlington, ON, Canada). 5,5′-dithiobis-(2-nitrobenzoic acid) (DTNB) was acquired from TCI Europe N.V. (Zwijndrecht, Belgium). *N,N*-Dimethylformamide (DMF) and acetonitrile (MeCN) were from VWR (Radnor, PA, USA). Chelex 100 resin was bought from Bio-Rad (Hercules, CA, USA). All of the experiments were performed in chelexed buffers and solutions. All buffers were prepared with Milli-Q water obtained with a deionizing water system from Merck KGaA (Darmstadt, Germany).

### 4.2. Peptide Synthesis

Model zinc peptides were synthesized using Fmoc solid phase synthesis (Fmoc-SPPS) using a Liberty 1 microwave-assisted synthesizer from CEM(Matthews, NC, USA) on a TentaGel R RAM Amide Rink resin of 0.2 mmol/g occupancy (RAPP Polymere GmbH, Tübingen, Germany) [15]. Peptides were N-terminally acetylated using acetic anhydride (Avantor Performance Materials Poland, Gliwice, Poland). Peptide cleavage from the resin was performed with a mixture of TFA/phenol/water/TIPS (88/5/5/2, *v*/*v*/*v*/*v*) over a period of 2 h followed by precipitation with prechilled (−70 °C) diethyl ether. The crude peptide was rinsed and centrifuged several times with cold diethyl ether, dried, and eventually purified on HPLC (Dionex Ultimate 3000, Thermo Fisher Scientific, Waltham, MA, USA) with Phenomenex C18 columns (Torrance, CA, USA). Peptide was separated from side products in a gradient of MeCN in 0.1% TFA/water from 0.5% to 50% over 30 min. The identity of each peptide was confirmed by an API 2000 ESI-MS spectrometer (Applied Biosystems, Waltham, MA, USA). The concentrations of the peptide stock solutions (in 5 mM HCl) were determined with Ellman’s reagent, using a molar absorption coefficient of 14,150 M^−1^ × cm^−1^ [66].

### 4.3. Expression and Purification of Metal-Free HsHk183

The production of *H. sapiens* Rad50 central fragment (*Hs*Hk183 and *Hs*Hk72) variants relied on the IMPACT Protein Purification System (NEB, Ipswich, MA, USA) with pTWIN1 vector and *E. coli* Bl21-CodonPlus (DE3)-RIL strain [67,68]. The phosphorylated HsHk183 was not expressed. Transformed bacterial cells were cultivated in 8 L of LB medium, grown at 37 °C until OD_600_ reached 0.45–0.55, then induced with 0.5 mM IPTG. Cultures were incubated for 5 h at 30 °C and shaking; cells were collected by centrifugation at 5000× *g* for 15 min at 4 °C. Pellets were resuspended in cold buffer A (50 mM Tris-Cl, pH 8.0 at 4 °C, 500 mM NaCl, 1 mM TCEP) and sonicated on ice for 30 min. Cell lysate was clarified by centrifugation at 20,000× *g* for 20 min. After overnight incubation of cell lysate with chitin resin with mild nutation, the chitin resin was washed with 20 bed volumes of buffer A with additional salt (1 M NaCl) to remove nonspecifically bound *E. coli* proteins. The intein-mediated cleavage reaction was initiated by buffer B with lower pH (50 mM Tris-HCl, pH 6.5 at 22 °C, 500 mM NaCl, 1 mM TCEP), which was added to the resin and incubated for 24 h at room temperature with mild shaking. Eluted protein was concentrated to a small volume using Amicon Ultra-10 Centrifugal Filter Units with a nominal molecular weight limit of 10 kDa (Merck Millipore, Burlington, MA, USA), followed by reverse-phase HPLC (Dionex Ultimate 3000, Thermo Fisher Scientific, Waltham, MA, USA) in a gradient of MeCN in 0.1% TFA/water, followed by lyophilization. The concentrations of the protein stock solutions (in 5 mM HCl) were determined with Ellman’s reagent.

### 4.4. XANES and EXAFS

X-ray absorption measurements (XANES and EXAFS) were performed at Zinc K-edge using double crystal monochromator [69] at the B18 beamline on Diamond Light Source. Spectra were collected with 0.3 eV steps in the energy range 9460–10,500 eV. Beforehand, *Hs*Hk183 variants were incubated overnight in Zn(II) buffering solution containing 4 mM ZnSO_4_ and 5 mM EGTA and purified via SEC on an ENrich SEC 70 10 × 300 column (Bio-Rad, Hercules, CA, USA). Samples were measured in 50% ethylene glycol 50 mM HEPES (pH 7.4, *I* = 150 mM coming from NaCl) 0.5 mM TCEP, after transferring to a gelatin capsule and freezing in liquid nitrogen. Sample measurement was conducted in cryostat set for −179.15 °C, in fluorescence mode with a 36-element germanium detector. Each acquired spectrum was inspected for glitches and nonlinear events before inclusion in the final averaged spectrum. A comparison of the first and the last scan for both samples showed no evidence of sample damage due to radiation. The final spectrum is an average of at least 60 scans. Data reduction, background subtraction, and fitting were performed with the modules of the Demeter suite [70] and IFEFFIT [71].

### 4.5. SAXS

SAXS measurements were carried out at the B21 beamline on Diamond Light Source. Samples were prepared by dissolving Rad50 fragment lyophilizate in water, then proteins were incubated overnight in Zn(II) buffering solutions of 50 mM chelators (triphosphate and HEDTA with 10 mM and 25 mM ZnSO_4_, respectively) in 50 mM HEPES, pH of 7.4, *I* = 0.1 from NaCl. After incubation, homogeneous fractions of Zn(II)-loaded proteins were collected via SEC on ENrich SEC 70 10 × 300 column (Bio-Rad, Hercules, CA, USA). Protein fractions were concentrated on Amicon Ultra-10 Centrifugal Filter Units before being injected on a KW402.5-4F Shodex size exclusion column (Showa, Denko, Tokyo, Japan) equilibrated with 50 mM HEPES, pH of 7.4, 100 mM NaCl). Data collection was conducted via a PILATUS 2M detector. For direct SAXS analysis, peak fractions were taken. All analysis steps were performed in Scatter (www.bioisis.net, accessed on 18 March 2022), followed by modeling via DENSS [35].

### 4.6. Spectroscopic Studies

#### 4.6.1. Electron Absorption Spectroscopy in UV Range

Absorbance spectra were recorded on a Jasco V-650 spectrophotometer (JASCO Corporation, Tokyo, Japan) in the spectral range of 200–320 nm in 1.0 cm cuvettes. The spectra were acquired for peptides in 20 mM Tris-HCl buffer, pH 7.4 (*I* = 0.1 coming from 0.1 M NaClO_4_), with 3-fold excess of TCEP over each cysteine in a peptide.

#### 4.6.2. Spectropolarimetric Titrations with Zn(II)

Titrations were performed at 25 °C by adding aliquots of ZnSO_4_ to a quartz cuvette (2 mm path length) containing peptide solution. Measurements were performed in 20 mM Tris-HCl buffer, pH 7.4 (*I* = 0.1 M from NaClO_4_), with a 5-fold excess of TCEP over each cysteine in the peptide or protein. Spectra were measured with the Jasco-1500 (JASCO Corporation, Tokyo, Japan); three accumulations were averaged to generate the final spectrum. Each spectrum was taken with 2 nm band width, 200 nm × min^−1^ scanning speed, and 1 nm data pitch.

### 4.7. Competitive Titrations

The apparent formation constants of Zn(II) biscomplexes Zn(*Hs*Hk)_2_ were determined spectropolarimetrically (Jasco J-1500, JASCO Corporation, Tokyo, Japan) at 25 °C, pH 7.4 in the presence of Zn(II) chelators CDTA (log*K* = 13.43), EDTA (log*K* = 12.64), HEDTA (log*K* = 11.18), EDDS (log*K* = 9.64), EGTA (log*K* = 8.2), and NTA (log*K* = 7.36) [9,44,72], according to previously established protocols [12,15]. In the case of proteins, 5–6 μM concentration was used. Proteins were incubated with 25 μM chelator in 20 mM Tris-Cl buffer (pH 7.4, *I* = 0.1 M from NaClO_4_) with 125 μM TCEP; afterwards, samples were titrated with ZnSO_4_ up to 22.5 μM concentration. CD signals for peptides were measured with 40–50 μM peptide concentrations and incubated with 500 μM chelators and titrated with ZnSO_4_ up to 450 μM concentration. After each ZnSO_4_ addition, the CD signal was measured until stabilized. Since EDDS is a weakly optical active chelator, a control titration with ZnSO4 was performed, and the signal of the peptides and proteins measured in the presence of EDDS was correlated with the control value. The free Zn(II) concentration for each ZnSO_4_ addition was calculated using the total chelator and ZnSO_4_ concentrations, corrected for the Zn(II) bound to the polypeptide (formation of Zn(*Hs*Hk)_2_ complex). Calculation of free Zn(II) in samples was performed using the Hyperquad Simulation and Speciation Software HySS 2009 [73]. In order to obtain the apparent formation constant, we normalized isotherms corresponding to complex formation by fitting with Hill’s equation. The obtained concentrations of free Zn(II), referring to the half-point complex saturation (${\left[\mathrm{Zn}\left(\mathrm{II}\right)\right]}_{\mathrm{free}}^{0.5}$) where half of the total peptide or protein is in the form of the Zn(*Hs*Hk)_2_ complex and half in the metal-free form, were used to calculate the apparent dissociation constants (*K*_12_) based on Equation (4), where *C_m_* is the total *Hs*Hk peptide concentration. Equation (4) is derived from Equation (5).

(4) $$\begin{array}{c} K_{12}=10^{\left(-\log{\left[\mathrm{Zn}\left(\mathrm{II}\right)\right]}_{\mathrm{free}}^{0.5}\right)}\times \left(\frac{C_{m}}{2}\right)\; \end{array}$$

(5) $$\begin{array}{c} K_{12}=\frac{\left[\mathrm{Zn}{\left(Hs\mathrm{Hk}\right)}_{2}\right]}{{\left[\mathrm{Zn}\left(\mathrm{II}\right)\right]}_{\mathrm{free}}{\left[Hs\mathrm{Hk}\right]}^{2}} \end{array}$$

### 4.8. Potentiometry

The protonation constants of the Hk6-Hk10 zinc hook peptides and stability constants of their Zn(II) complexes were determined at 25 °C at 0.1 M ionic strength (from KNO_3_) by pH-metric titration over a range of 2.8 to 10.8 using a automatic titrator (Molspin Ltd., Newcastle upon Tyne, United Kingdom) under an argon atmosphere with standardized 0.1 M NaOH as a titrant. The data were analyzed and plotted using SUPERQUAD [74] and HySS software [73]. The *K*_12_ values were calculated as in a previous study [15].

## Figures and Tables

**Table 1:** Conditional formation constants (log*K*_12_) of Zn(*Hs*Hk)_2_ of human zinc hook peptides, their mutants, and phosphorylated variants at pH 7.4, *I* = 0.1 M, 25 °C. Values were determined by competition experiments with zinc chelators with spectropolarimetric detection or calculated from cumulative protonation and stability constants determined by potentiometry.

Zinc Hook Peptide	log*K*_12_–Competition	log*K*_12_–Potentiometry
*Hs*Hk4	n.d. ^a^	14.93 ^b^
*Hs*Hk6	n.d.	15.13
*Hs*Hk10	16.5 ± 0.4	16.15
*Hs*Hk14	15.8 ± 0.4	n.d.
*Hs*Hk42	17.26 ± 0.08	n.d.
*Hs*Hk42 pT690	16.4 ± 0.2	n.d.
*Hs*Hk42 T690E	16.5 ± 0.1	n.d.
*Hs*Hk72	18.3 ± 0.4	n.d.
*Hs*Hk140	19.5 ± 0.2	n.d.
*Hs*Hk183	19.53 ± 0.06	n.d.
*Hs*Hk183 T690E	19.27 ± 0.05	n.d.

^a^ n.d.—not determined; ^b^ constant taken from [15].

**Table 2:** Conditional formation constants for selected Zn(II) binding proteins or peptides with details of experimental conditions found across the literature. Some of the presented polypeptides form complexes of stoichiometry 1:2 Zn(II):polypeptide, which is indicated.

Protein Complex	Residues in Coordination Sphere (Complex Stoichiometry)	Experimental Conditions	log*K* ^a^	log*K* Estimated to pH 7.4 ^b^	CI ^c^ Estimated to pH 7.4 ^b^	Reference
CD4-Lck kinase complex (*H. sapiens*)	2 × CC (ZnL′L″)	150 mM NaCl, 10 mM Na_2_SO_4_, 0.08%, β-ME, 50 mM ZnSO_4_, 10 mM HEPES, pH 7.0	6.4	8	4.7	[51]
CD4-Lck kinase complex (*H. sapiens*)	2 × CC (ZnL′L″)	20 mM Tris, 10 mM NaClO_4_, 15 μM TCEP, pH 7.4	18.97	18.97	15.67	[52]
CD8α-Lck kinase complex (*H. sapiens*)	2 × CC (ZnL′L″)	150 mM NaCl, 10 mM Na_2_SO_4_, 0.08%, β-ME, 50 mM ZnSO_4_, 10 mM HEPES, pH 7.0	6.05	7.65	4.35	[51]
Rad50 (*P. furiosus*)	2 × CC (ZnL_2_)	20 mM Tris-HCl, 100 mM NaClO_4_, pH 7.4	20.82	20.82	17.52	[27]
Rad50 (*H. sapiens*)	2 × CC (ZnL_2_)	20 mM Tris-HCl, 100 mM NaClO_4_, pH 7.4	19.52	19.52	16.22	this study
CP1	CCCC (ZnL)	50 mM HEPES, 100 mM NaCl, pH 7.0	12.0	13.6	13.6	[60]
CP1	CCHH (ZnL)	50 mM HEPES, 100 mM NaCl, pH 7.0	11.2	12	12	[60]
MTF1-1 (*H. sapiens*)	CCHH (ZnL)	50 mM HEPES, pH 7.4, 100 mM NaClO_4_	11.6	11.6	11.6	[61]
MTF1-1 (*H. sapiens*)	CCHH (ZnL)	100 mM HEPES, 50 mM NaCl, pH 7.0	10.5	11.3	11.3	[62]
PDLIM1 (*H. sapiens*)	CCCC (ZnL)	50 mM Tris, 100 mM NaCl, pH 7.4	14.5	14.5	14.5	[49]

^a^ *K*_a_ is a conditional formation constant; in the case of intramolecular complexes *K*_1_ is given, in the case of intermolecular dimeric complexes *K*_12_ is given, as defined in Equations (1)–(3). ^b^ Estimation of values at pH 7.4 is done by applying a correction factor of 0.1 of log*K*_a_ value per 0.1 pH unit counted per one cysteine residue in the coordination sphere. ^c^ CI is defined as −log*K*_a_ of the theoretical ZnL complex where [ZnZ] = Σ*_ijk_*[Zn*_i_*H*_j_*L*_k_*] and *K*_a_ =[Zn] × [Z] × [ZnZ]^−1^. The concentrations of Zn(II) and L were set at 0.25 mM and 1 mM, respectively. β-ME—β-mercaptoethanol; L′L″—heterodimer; L_2_—homodimer.

## Data Availability

Data is contained within the article.

## Supplementary Materials

The supporting information can be downloaded at: https://www.mdpi.com/article/10.3390/ijms231911140/s1. Reference [75] is cited in the supplementary materials.

## Abbreviations

ATM	ataxia-telangiectasia mutated kinase
CD	circular dichroism
CI	competitivity index
EXAFS	extended X-ray absorption fine structure
*Hs*Hk	zinc hook from *H. sapiens* Rad50
MRN	Mre11, Rad50, Nbs1 complex
NHEJ	non-homologous end joining
PDB	Protein Data Bank
*Pf*Hk	zinc hook from *P. furiosus* Rad50
SAXS	Small angle X-ray scattering
XANES	X-ray absorption near-edge structure
