# Supplementary material for: Relations between Structure and Zn(II) Binding Affinity Shed Light on the Mechanisms of Rad50 Hook Domain Functioning and Its Phosphorylation
Source: Int J Mol Sci. 2022 Sep 22;23(19):11140. doi: 10.3390/ijms231911140 (PMC9569753; doi:10.3390/ijms231911140)
Supplement: Supplementary file 1 [file ijms-23-11140-s001.zip › ijms-1902710-supplementary.pdf]

Supporting Information

Relations between structure and Zn(II) binding affinity shed light on the mechanisms of Rad50 hook domain functioning and its phosphorylation

Józef Ba Tran, Michał Padjasek and Artur Krężel *

Department of Chemical Biology, Faculty of Biotechnology, University of Wrocław, Joliot-Curie 14a, 50-383 Wrocław, Poland
* Correspondence: artur.krezel@uwr.edu.pl

Table S1. Sequences and corresponding theoretical and experimental molecular mass values of synthesized and overexpressed peptides and proteins. m and av refer to monoisotopic and averaged mass, respectively. Ac-, $-NH_2$ denote acetyl and amide groups on N- and C-terminus, respectively. pT denotes phosphothreonine. Additional residues or mutations and modifications not existing in the wild-type sequence are bolded.

Peptide name	Peptide sequence	Calculated molecular weight	Measured molecular weight
HsHk4	Ac-CPVC-NH_2	461.2^{m}	461.3^{m}
HsHk6	Ac-CPVCQR-NH_2	745.3^{m}	745.6^{m}
HsHk10	Ac-SCCPVCQRVF-NH_2	1181.5^{m}	1182.3^{m}
HsHk14	Ac-NQSCCPVCQRVFQT-NH_2	1652.7^{av}	1653.8^{m}
HsHk42	Ac-VYSQFITQLTDENQSCCPVCQRVFQTEAELQEVISDLQSKLR-NH_2	4919.5^{av}	4920.1^{av}
HsHk42 pT690	Ac-VYSQFITQLTDENQSCCPVCQRVFQ**pT**EAELQEVISDLQSKLR-NH_2	4999.5^{av}	5000.1^{av}
HsHk42 T690E	Ac-VYSQFITQLTDENQSCCPVCQRVFQ**E**EAELQEVISDLQSKLR-NH_2	4947.5^{av}	4958.0^{av}
HsHk72	KSSKQRAMLAGATAVYSQFITQLTDENQSCCPVCQRVFQTEAELQEVISDLQSKLRLAPDKLKSTESELKK	7949.1^{av}	7949.9^{av}
HsHk140	**C**NELKRKEEQLSSYEDKLFDVCGSQDFESDLDRLKEEIEKSSKQRAMLAGATAVYSQFITQLTDENQSCCPVCQRVFQTEAELQEVISDLQSKLRLAPDKLKSTESELKKKEKRRDEMLGLVPMRQSIIDLKEKEIPELR	16233.5^{av}	16233.9^{av}
HsHk183	**C**KEINQTRDRLAKLNKELASSEQNKNHINNELKRKEEQLSSYEDKLFDVCGSQDFESDLDRLKEEIEKSSKQRAMLAGATAVYSQFITQLTDENQSCCPVCQRVFQTEAELQEVISDLQSKLRLAPDKLKSTESELKKKEKRRDEMLGLVPMRQSIIDLKEKEIPELRNKLQNVNRDIQRLKN	21343.2^{av}	21343.8^{av}
HsKk183 T690E	**C**KEINQTRDRLAKLNKELASSEQNKNHINNELKRKEEQLSSYEDKLFDVCGSQDFESDLDRLKEEIEKSSKQRAMLAGATAVYSQFITQLTDENQSCCPVCQRVFQ**E**EAELQEVISDLQSKLRLAPDKLKSTESELKKKEKRRDEMLGLVPMRQSIIDLKEKEIPELRNKLQNVNRDIQRLKN	21371.2^{av}	21371.6^{av}

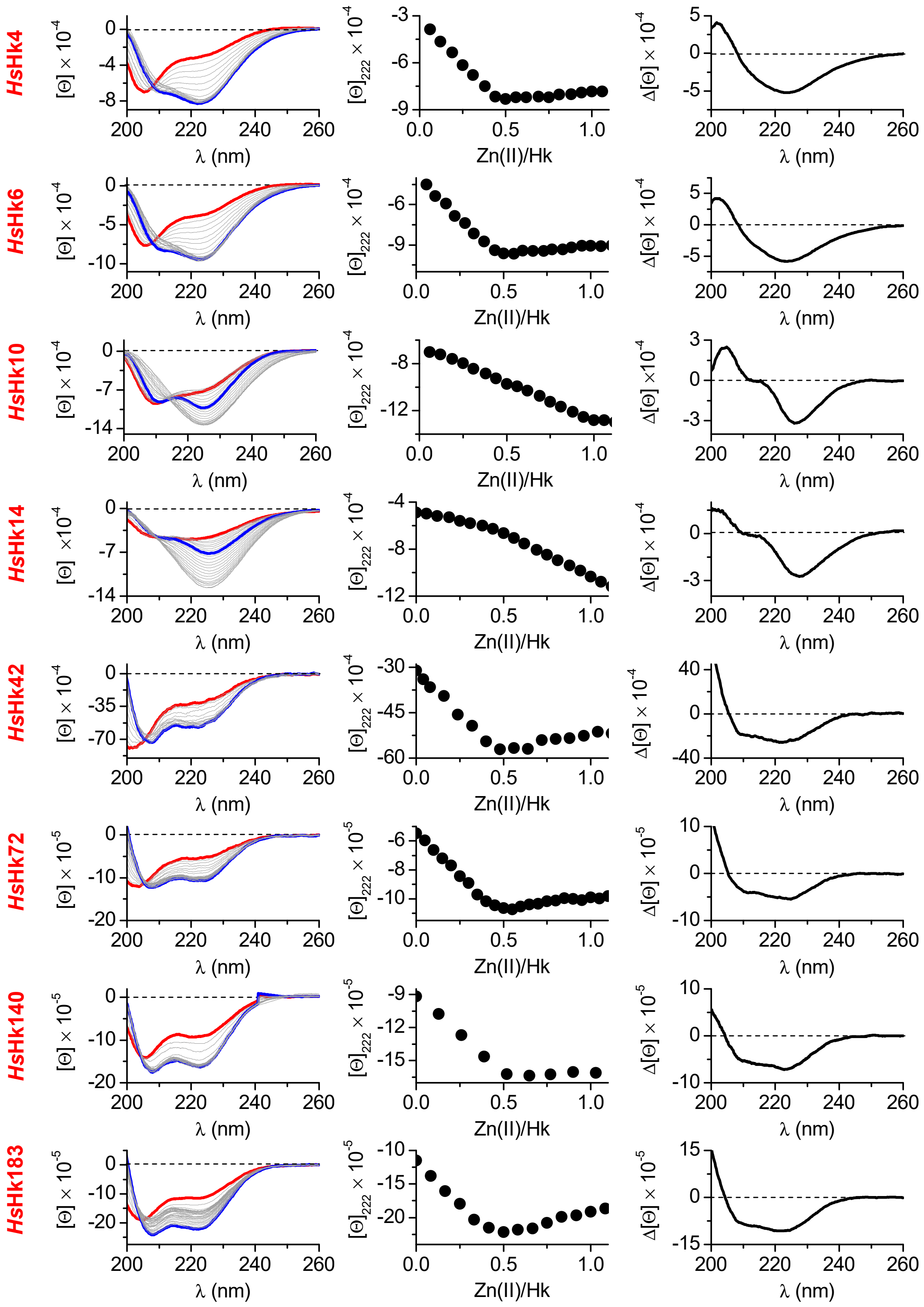

Figure S1. CD spectra of wild-type *Hs*Hk4-182 titrated with Zn(II). Panel on the left-hand side shows molar ellipticity changes (in deg × cm^2 × $dmol^{-1}$) in the function of wavelength; red line represents apo-form of Rad50 peptides, blue line represents 0.5 Zn(II) molar equivalent over Rad50 peptide. The middle panel shows molar ellipticity

changes at 220 nm versus Zn(II)/HsHk molar ratio. The right-hand side panel shows differential CD spectra of HsHk peptides at the Zn(II)/HsHk molar ratio of 0.5.

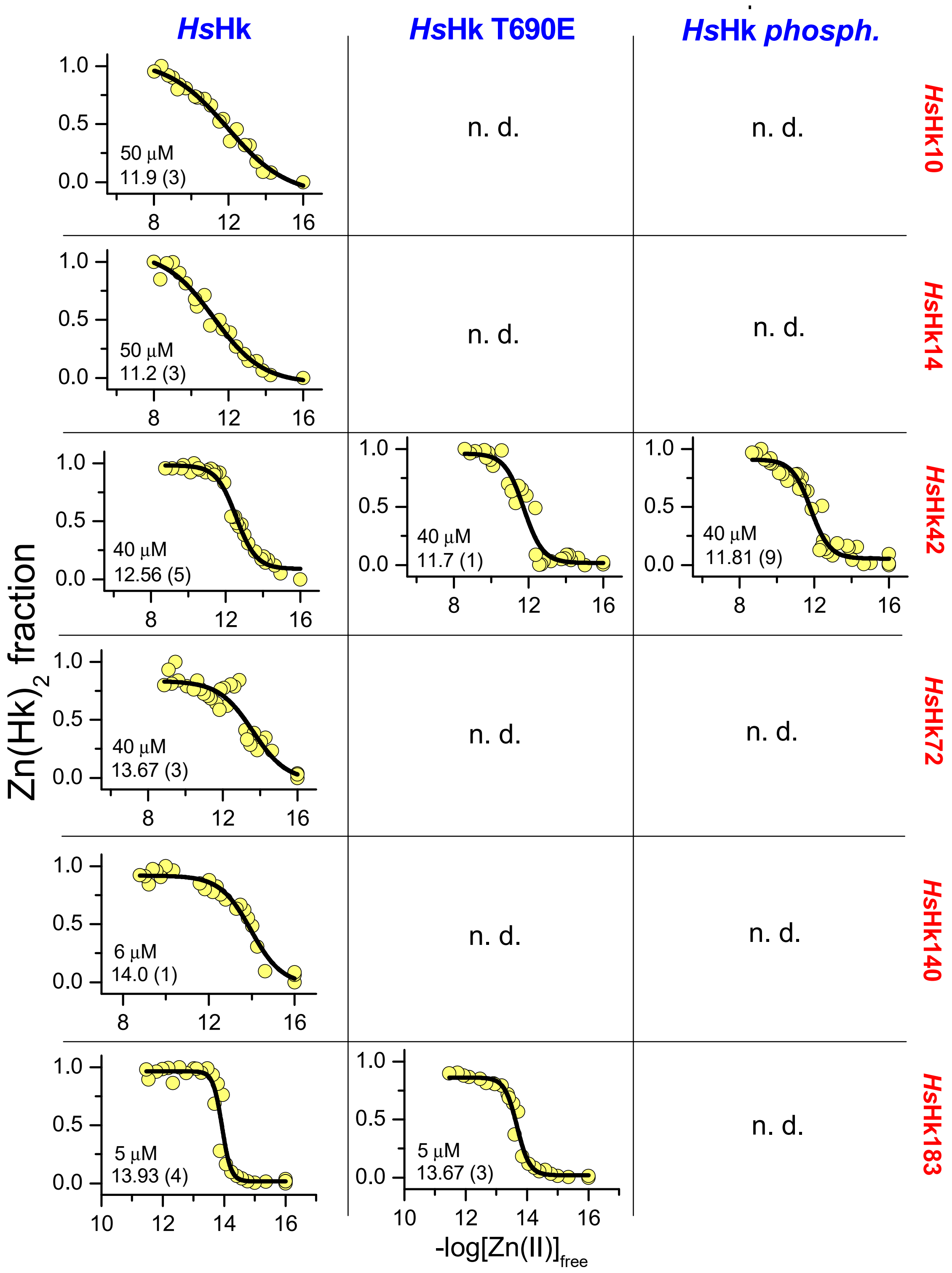

Figure S2. Isotherms Zn(II) binding to *Hs*Hk10-183 in the presence of a five-fold molar excess of metal chelators. The columns from left to right show the competitive titrations of wild-type, phosphomimetic mutant and phosphorylated *Hs*Hk constructs. Data were acquired spectropolarimetrically in 20 mM Tris-HCl buffer with 100 mM

NaF, pH 7.4. Peptides were used at various concentrations that are listed in the graphs. Data were fitted to the Hill equation; the presented values correspond to the half-saturation points (-log$[Zn(II)]_{free}^{0.5}$). The half-saturation points were used for calculating K_{12} as described in Materials and methods.

Table S2. Cumulative protonation and Zn(II) stability constants ($\log\beta_{ijk}$)[a] of hook peptide complexes determined potentiometrically at 25°C, I = 0.1 M (from KNO_3).[b]

Species	*Hs*Hk6	*Hs*Hk10
HL	9.09 ± 0.04	9.45 ± 0.05
H_2L	16.69 ± 0.03	18.26 ± 0.02
H_3L	-	25.35 ± 0.04
ZnL	9.82 ± 0.06	19.32 ± 0.09
ZnH_2L_2	-	37.85 ± 0.07
$ZnHL_2$	-	30.74 ± 0.05
ZnL_2	19.34 ± 0.07	21.35 ± 0.08

[a] β $M_iH_jL_k$ = $[M_iH_jL_k]/([M]^i[H]^j[L]^k)$, where [L] is the concentration of fully deprotonated zinc hook peptide.[b] Standard deviations are given as provided by SUPERQUAD calculations.

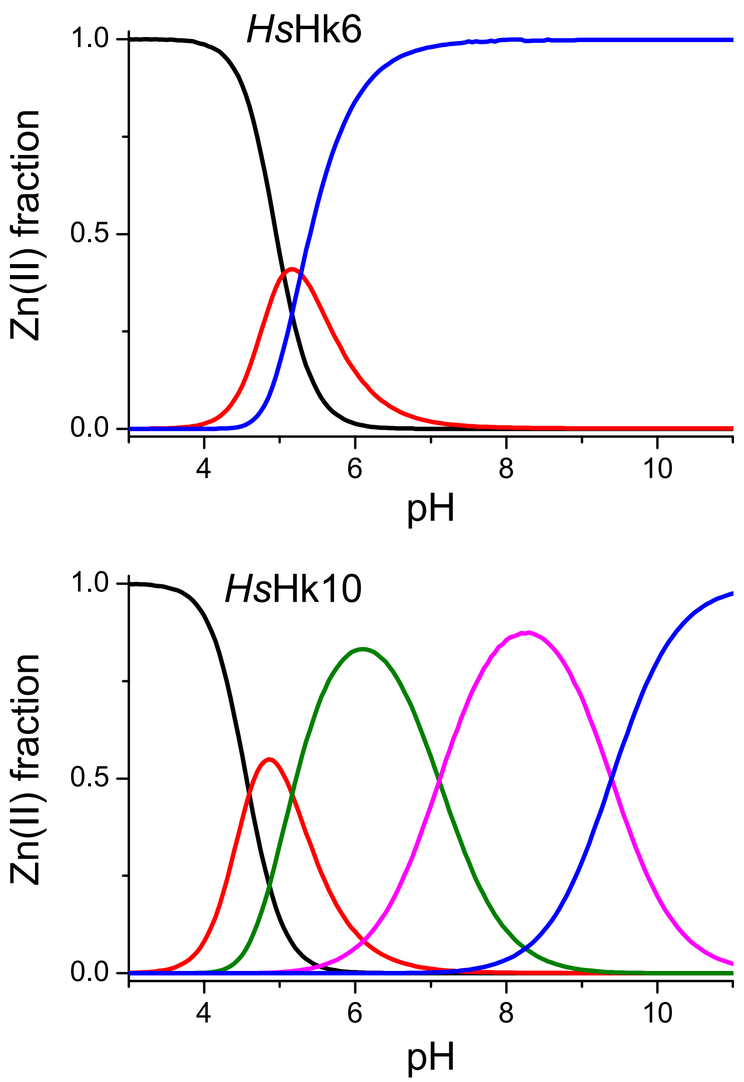

Figure S3. Molar species distribution of Zn(II) complexes with *Hs*Hk6 and *Hs*Hk10 peptides calculated based on protonation and stability constants determined potentiometrically (see Table S2). *Hs*Hk peptides and metal ion concentrations were set as 1.0 and 0.5 mM, respectively, as in potentiometry experiments. Black, red, olive, magenta and blue lines stand for free Zn(II), ZnL, ZnH_2L_2, $ZnHL_2$, and ZnL_2, respectively. *Hs*Hk6 does not form ZnH_2L_2 and $ZnHL_2$, no lines representing those species are graphed.

Table S3. Parameters used to simulate the EXAFS for zinc K edge of WT HsHk183 Rad50 fragment and T690E HsHk183 Rad50 fragment. N is the degeneracy of the path, S^2_0 is an amplitude factor, σ^2 is a Debye-Waller-like factor equivalent to the mean square deviation of an atom from its mean position, ΔE_0 is an energy shift interpreted as the alignment of the energy grids of the data and theory, ΔR is an adjustment to the half path length, R_{eff} is the initial half path length, R is fitted distance of atoms from the Zn(II).

	WT			T690E		
element	sulfur	sulfur	carbon	sulfur	sulfur	carbon
N	3	1	4	3	1	4
S^2_0	1.14	1.14	1.14	1.08	1.08	1.08
σ^2 [Å^2]	5.17×10^{-3}	5.17×10^{-3}	1.22×10^{-2}	4×10^{-3}	4×10^{-3}	1.14×10^{-2}
ΔE_0 [eV]	6.61	6.61	6.61	6.65	6.65	6.65
ΔR [Å]	0.12	0.12	-0.09	0.13	0.13	-0.09
R_{eff} [Å]	2.20	2.22	3.24	2.20	2.22	3.24
R [Å]	2.32	2.35	3.14	2.32	2.35	3.15

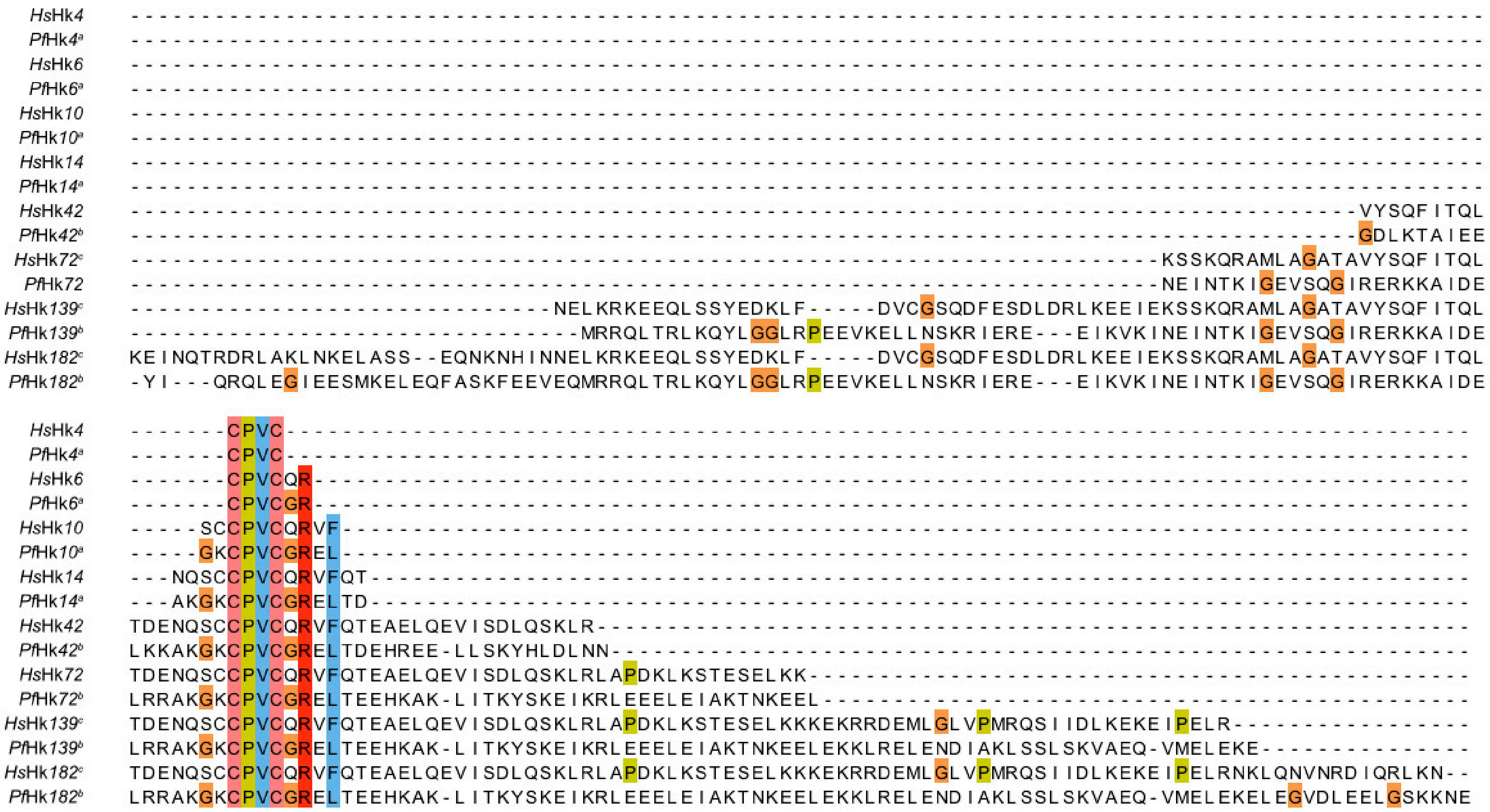

Figure S4. Protein sequence alignment of zinc hook domains (up to 182 amino-acid long) from *P. furiosus* (*Pf*Hk) and *H.sapiens* (*Hs*Hk). The compared *Pf*Hk fragments are selected to have a the same length of the CPVC-flanking regions as in the *Hs*Hk constructs. Alignment was made with Clustal Omega using default parameters [1]. [a] *P. furiosus* fragments used in [2]. [b] The fragment has the same length as the compared fragment from *H. sapiens* Rad50. [c] Presented fragment has no additional N-terminal cysteine that was introduced in the construct used in the protein expression.

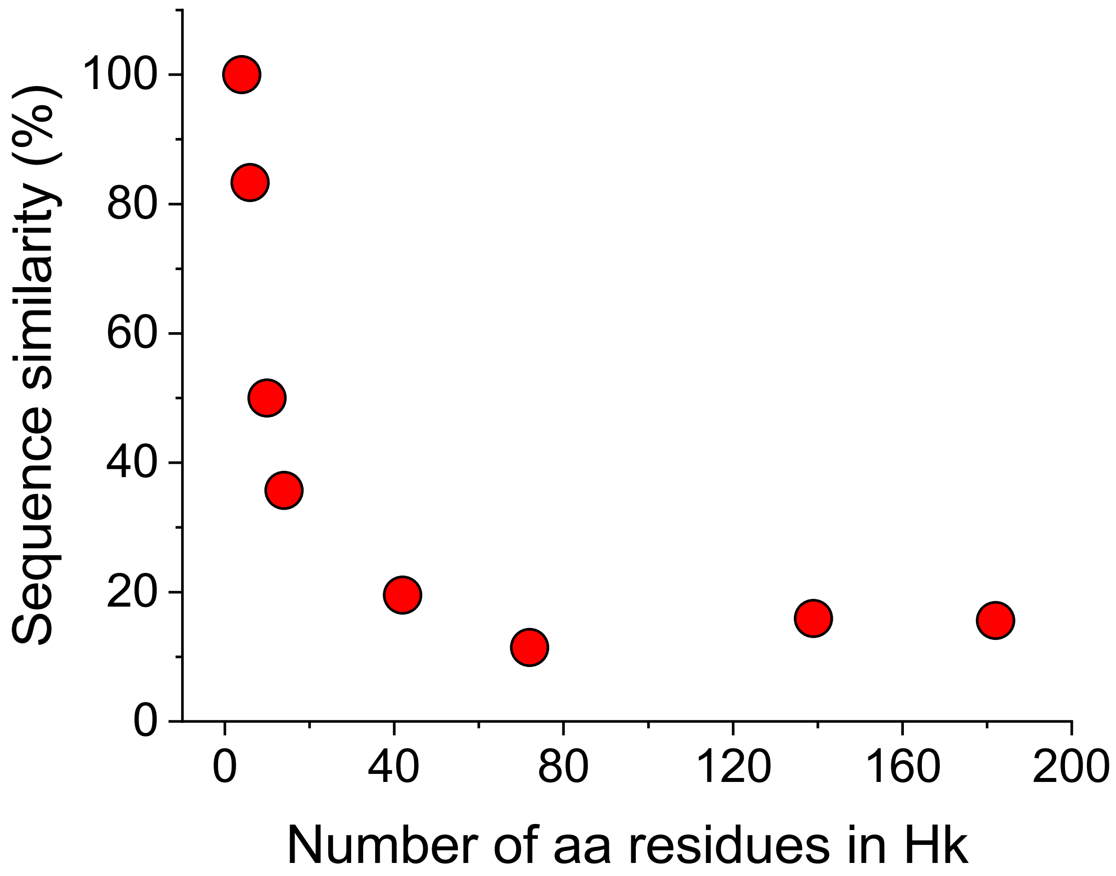

Figure S5. Sequence similarity between the zinc hook domain fragments from *P. furiosus* and *H.sapiens* (Figure S5). Percent identiy has been calculated with Clustal Omega using default parameters [1].

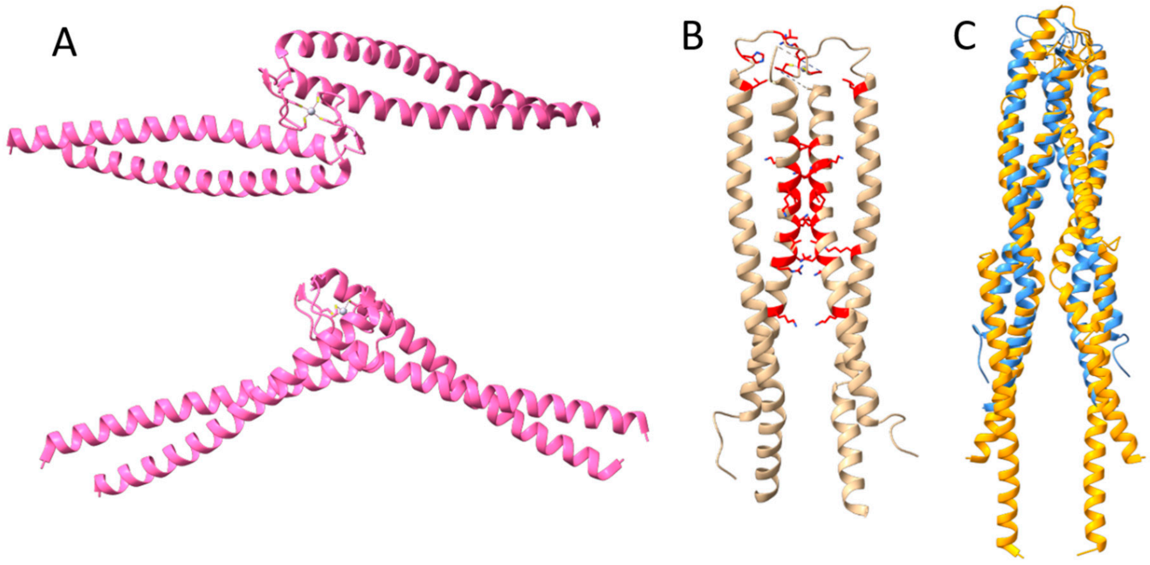

Figure S6. (a) The open structure of *P. furiosus* Rad50 (PDB ID 1L8D) in cartoon representation. (b) In cartoon representation, the rod-shaped structure of *P. furiosus* Rad50 (PDB: 6ZFF). Residues marked in red are involved in the formation of the interface. Except for the not fully resolved zinc hook, the intermolecular interface

is formed at a distance of around 20 Å from the Zn(II). (c) Superposition of *H. sapiens* (PDB ID 5GOX) and rod-shaped *P. furiosus* Rad50 (PDB ID 6ZFF), presented respectively in orange and light blue. Human Rad50 zinc hook shows a non-exisstent interface in *P. furiosus* Rad50 located around 60 Å from the Zn(II).

References

1. Madeira, F.; Pearce, M.; Tivey, A.R.N.; Basutkar, P.; Lee, J.; Edbali, O.; Madhusoodanan, N.; Kolesnikov, A.; Lopez, R. Search and Sequence Analysis Tools Services from EMBL-EBI in 2022. *Nucleic Acids Res.* **2022**, *50,* W276–W279. doi:10.1093/nar/gkac240.

2. Kochańczyk, T.; Nowakowski, M.; Wojewska, D.; Kocyła, A.; Ejchart, A.; Koźmiński, W.; Krężel, A. Metal-Coupled Folding as the Driving Force for the Extreme Stability of Rad50 Zinc Hook Dimer Assembly. *Sci. Rep.* **2016**, *6*, 36346. doi:10.1038/srep36346.
